# Multilevel Nonlinear Mixed-Effect Crown Ratio Models for Individual Trees of Mongolian Oak (*Quercus mongolica*) in Northeast China

**DOI:** 10.1371/journal.pone.0133294

**Published:** 2015-08-04

**Authors:** Liyong Fu, Huiru Zhang, Jun Lu, Hao Zang, Minghua Lou, Guangxing Wang

**Affiliations:** 1 Research Institute of Forest Resource Information Techniques, Chinese Academy of Forestry, Beijing, 100091, P. R. of China; 2 Research Center of Forestry Remote Sensing and Information Engineering, Central South University of Forestry and Technology, Changsha, 410004, Hunan, P. R. of China; 3 Department of Geography and Environmental Resources, Southern Illinois University at Carbondale, Carbondale, Illinois, 62901, United States of America; Tennessee State University, UNITED STATES

## Abstract

In this study, an individual tree crown ratio (CR) model was developed with a data set from a total of 3134 Mongolian oak (*Quercus mongolica*) trees within 112 sample plots allocated in Wangqing Forest Bureau of northeast China. Because of high correlation among the observations taken from the same sampling plots, the random effects at levels of both blocks defined as stands that have different site conditions and plots were taken into account to develop a nested two-level nonlinear mixed-effect model. Various stand and tree characteristics were assessed to explore their contributions to improvement of model prediction. Diameter at breast height, plot dominant tree height and plot dominant tree diameter were found to be significant predictors. Exponential model with plot dominant tree height as a predictor had a stronger ability to account for the heteroskedasticity. When random effects were modeled at block level alone, the correlations among the residuals remained significant. These correlations were successfully reduced when random effects were modeled at both block and plot levels. The random effects from the interaction of blocks and sample plots on tree CR were substantially large. The model that took into account both the block effect and the interaction of blocks and sample plots had higher prediction accuracy than the one with the block effect and population average considered alone. Introducing stand density into the model through dummy variables could further improve its prediction. This implied that the developed method for developing tree CR models of Mongolian oak is promising and can be applied to similar studies for other tree species.

## Introduction

Tree crown size is an important variable that is commonly involved in growth and yield models used as decision-support tools in forest management [1–3]. Tree crown size is usually characterized using crown length (CL), crown width (CW), and crown ratio (CR). Tree CR is defined as the percentage of crown length from the base of live crown to the tree top to its total height. The value ranges from 0 (for the trees without crown, such as dead or defoliated trees) to 1 (for the trees with crowns extending the entire tree bole). Tree CR is considered to be an indirect measure of photosynthetic capacity of trees [2]. Moreover, tree CR is also a useful indicator of vigor [4–7] and stand density [8]. Additionally, it is a variable of interest in the management of many non-timber resources including recreation forests and wildlife habitats [9]. In forest inventory, however, measuring tree CR for all sampled trees is time- and money-consuming (e.g., [5, 10]). Therefore, developing accurate tree CR models is necessary, which allows forest managers to accurately predict tree CR.

One common approach used to obtain values of tree CR is to develop either deterministic or stochastic models from tree characteristics [1–3, 5, 10, 11]. So far, most of the obtained models (see Table 1) are simply linear or nonlinear (e.g., Exponential, logistic and Weibull models) and often developed as a function of diameter at breast height (1.3 m above ground) (D), total tree height (H), age (A) and so on, using ordinary least squares techniques [12, 13]. When the relationships of tree CR with other tree variables are modeled, measurements of the variables are often collected within sample plots that are allocated in different stands that represent different site conditions, called blocks in this study). This hierarchical structure leads to the fact that the selected trees are located within the same plots and the plots selected within the same blocks and that measurements are likely correlated with each other significantly [14–16]. In this case, using ordinary least square regression technique to fit the data often leads to bias as the standard errors of parameter estimates would get inflated [15]. To deal with this problem, several approaches have been proposed [14, 17, 18].

**Table 1:** Existing tree crown ratio (CR) models.

Model	Predictors	Model form	Source	Model no
*CR* = [1 + exp(**βX**)]^-1^	HT, D, HT/D,BAL, CCF, ELEV,SL,AZ	Logistic	Hasenauer and Monserud, 1996	II.1
*CR* = [1 + exp(**βX**)]^-1^	D,HT,TSC,CCF,BAL, ELEV, SL, ASPECT	Logistic	Temesgen et al., 2005	II.1
*CR* = [1 + exp(**βX**)]^-1^	CCR, D	Logistic	Toney and Reeves, 2008	II.1
*CR* = [1 + exp(**βX**)]^-1^	D, HT, BA	Logistic	Leites et al., 2009	II.1
*CR* = [1 − exp(**βX**)]^-1^	MHT, TSC	Logistic	Popoola and Adesoye, 2012	II.2
*CR* = *a*/[1 + *b* exp(−**βX**)]	Age, SD, DH, D	Logistic	Soares and Tomé, 2001	II.3
*CR* = *a* + exp(−**βX**)	MHT, TSC	Exponential	Popoola and Adesoye, 2012	II.4
*CR* = exp(**βX**)	D, HT, BA, CCF,PCT	Exponential	Leites et al., 2009	II.5
*CR* = *a*[1 – *b* exp(−**βX**)]	Age, SD, DH, D	Exponential	Soares and Tomé, 2001	II.6
*CR* = [1 − exp(−*ϕ* **βX**)]	Age, D, HT,	Exponential	Dyer and Burkhart, 1987	II.7
*CR* = [1 − exp(−*ϕ* **βX**)]	BA, DH, D, HT	Exponential	Hynynen, 1995	II.7
*CR* = [1 + exp(−**βX**)]^−1/2^	MHT, TSC	Richards	Popoola and Adesoye, 2012	II.8
*CR* = *a*/[1 + *b* exp(−**βX**)]^1/*c*^	Age, SD, DH, D	Richards	Soares and Tomé, 2001	II.9
*CR* = *a*/[1 − *b* exp(−**βX** ^*c*^)]	Age, SD, DH, D	Weibull	Soares and Tomé, 2001	II.10

Note: MHT: merchantable height, TSC: tree slenderness coefficient, D: diameter at breast height, H: total tree height, BA: stand basal area, CCF: stand crown competition factor, PCT: The percentile in the stand basal area distribution, CCR: compacted crown ratio, BAL: basal area per ha for trees larger than the subject tree, ELEV: elevation, SL: slope, ASPECT: aspect, SD: stand density, DH: dominant tree height, AZ: azimuth of aspect in radians, Age: tree age, *a*, *b*, *c*: model parameters, **β**: parameter vector, x: vector of stand or tree variables.

One of the approaches is the use of nonlinear mixed-effect (NLME) models [19]. This approach provides an efficient way to analyze correlated hierarchical structure data and make accurate local predictions ([19, 20]. NLME models contain both fixed- and random-effect parameters. The fixed parameters provide the potential to account for covariate or treatment effects as in traditional regression, while the random parameters offer the capacity to explain various sources of heterogeneity and randomness in the data caused by known and unknown factors [20–25]. Therefore, the NLME models have been widely applied in forest growth and yield modelling over the past decades (e.g., [21–25]). They can be used not only to account for stochastic variability in tree CR models, but also to provide the potential of increasing accuracy of tree CR prediction. To the authors’ knowledge, however, only a few studies have been conducted to model tree CR using the NLME models, especially multilevel NLME models [26, 27].

The objective of this study was to develop an individual tree CR model for Mongolian oak (*Quercus mongolica*) natural forests where sample plots were selected and the trees within the plots were measured. A two-level NLME CR model with random effects at both block and plot levels was first built to account for autocorrelation of hierarchical structure data. Various tree and stand characteristics were then tested for their contributions to the improvement of the CR model fits and predictions. The predictive ability and applicability of the obtained NLME CR model were validated and demonstrated using an independent data set.

## Materials and Methods

### Study area and data

**Fig 1:**
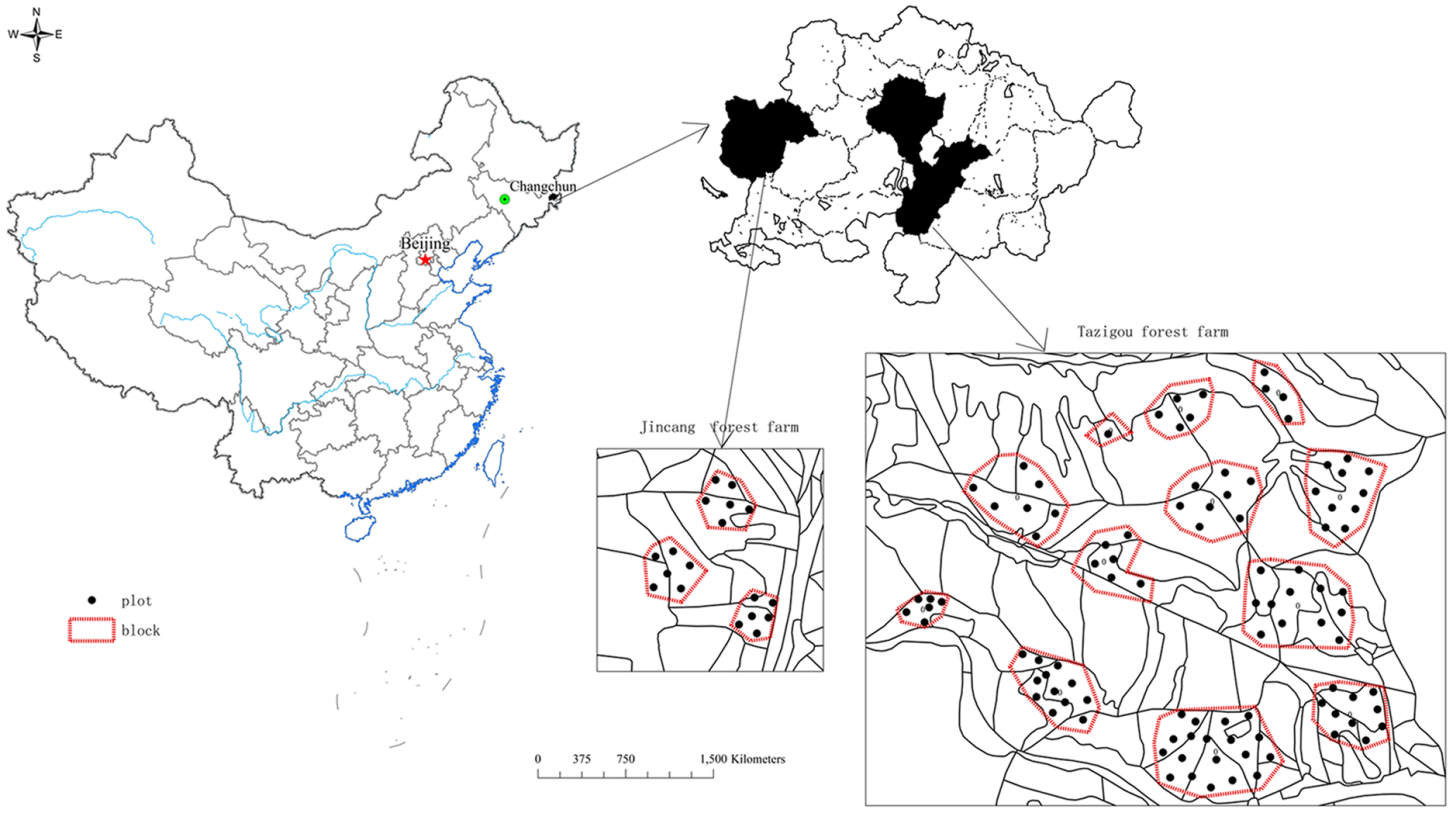
Location (upper left) of study area: Wangqing Forest Bureau (upper right) in northeast China and spatial distribution of 15 blocks and 118 sample plots (bottom).

The used data were obtained from a total of 118 permanent sample plots (PSPs) established in Mongolian oak natural forests allocated in Wangqing Forest Bureau of northeast China (123°56′–131°04′ E, 43°05′–43°40′ N) (Fig 1, Table 2). These PSPs were nested within 15 blocks, out of which 12 blocks with 100 PSPs are located in Tazigou forest farm and the other 3 blocks with 18 PSPs in Jincang forest farm, that were randomly located in the study area to represent the stands with different site conditions. When the data were collected, there were no regulations setup to limit the scientific research in this forestry bureau and thus no specific permission was required for the field work. In addition, there were no any endangered or protected species involved in the field study.

**Table 2:** Numbers of sample plots and trees grouped into classes of stand density index (SDI) for Mongolian oak.

Variable	Class	Class midpoint	Class range	Number of plots	Number of trees
Stand density index	1	150	0 < *SDI* ≤ 300	17	253
(trees ha^-1^)	2	350	300 < *SDI* ≤ 400	40	705
	3	450	400 < *SDI* ≤ 500	29	554
	4	550	500 < *SDI* ≤ 600	18	916
	5	600	*SDI* > 600	8	706

All the PSPs established in 2010 (100 plots) and 2013 (18 plots) by the Research Institute of Forest Resources Information Techniques, Chinese Academy of Forestry, were square in shape with an average size of 719 m^2^ and varying from 400 to 2500 m^2^. All standing live trees (H > 1.3 m and D > 5cm) within each of the plots were measured for D, H and trunk height to the crown base (HCB). Tree HCB was defined as the height from the ground to the base of the first normal green branch as a part of the crown; this excluded the secondary branches (epicormic and adventitious) [5]. Furthermore, a single green branch was not the base of tree crown if there were at least three dead whorls above it [5]. Three to five dominant or sub-dominant trees within each plot were chosen to measure plot dominant tree height (DH) and dominant tree diameter at breast height (DD) [28]. Stand age (A) was determined by the mean values of three average sample trees in each plot [29]. Canopy density (CD) for each plot was obtained using a moose-horn method [30]. The measurements of the tree variables were collected from a total of 3685 trees (Table 2).

Stand density (SD) affects tree growth significantly [3, 31, 32]. Our preliminary analyses revealed that the differences of tree CR among different SD values of the PSPs were significant at a risk level of 0.05. All the PSPs were thus stratified into five stand density classes by stand density index (SDI) [33] (Table 2) and the effect of SDI on tree CR was then analyzed based on the inventory data. The expression of SDI is given as [33]:

$$SDI=SD{\left(MD/D_{0}\right)}^{\beta }+\varepsilon$$

where *SD* is stand density (trees ha^-1^), *MD* is stand arithmetic mean diameter (cm), *D*_0_ is standard mean diameter (cm), *β* is an estimated parameter, and *ε* is an error term. The values of *D*_0_(*D*_0_ = 20) and *β* (*β* = 1.3798) in this study were obtained based on the study by Du et al. (2000) in which stand density index models for 11 tree species groups for Wangqing Forest Bureau, including Mongolian oak, were developed. The effect of Meyer’ site index (Meyer’ SI) on tree CR was also analyzed in this study. The Meyer’ SI was given by Du et al. [29]:

$$SI=DH\text{exp}\left[\left(\alpha /A)-(\alpha /A_{0}\right)\right]+\varepsilon$$

where *α* is a parameter, *A* is stand age (years) at time when DH measurements were collected, and *A*_0_ is the standard age (base age) (years) of SI, equal to 40 for Mongolian oak [29]. A multivariate analysis was carried out to detect outlier data based on the distribution of Mahalanobis distance between the observations and their expectations [15]. Only 6 PSPs were rejected from this technique as they represented special cases, namely very low dense stands or over mature stands. The remaining 112 PSPs were randomly divided into two groups: one for model fitting and the other for model validation. The data used for model fitting consisted of 2166 trees from 74 PSPs and the data for model validation was composed of 968 trees from 38 PSPs (Table A in S1 File). Summary statistics of the data and relevant stand characteristics are listed in Table 3.

**Table 3:** Summary statistics of stand variables from the sample plots used for model fitting and validation, respectively.

Data	Variable	Min	Max	Mean	SD	CV%
Plots for model fitting						
	Area (m^2^)	400	2500	719	581	80.83
	CR	0.06	1.00	0.61	0.17	28.49
	HCB (m)	0.40	13.30	4.60	2.39	51.86
	D (cm)	1.40	70.10	15.42	8.40	54.49
	H (m)	1.80	25.60	12.18	4.18	34.29
	SD (trees ha^-1^)	275	1863	877	515	59
	CD	0.46	0.90	0.79	0.09	10.97
	DH (m)	12.54	23.78	17.04	2.57	15.06
	DD (cm)	16.75	38.90	24.53	5.08	20.69
Plots for model validation						
	Area (m^2^)	0.04	0.25	0.08	0.07	92.32
	CR	0.01	0.93	0.60	0.18	29.15
	HCB (m)	0.50	14.00	4.76	2.43	51.04
	D (cm)	1.50	48.20	16.48	8.65	52.50
	H (m)	2.20	25.60	12.49	4.14	33.16
	SD (trees ha^-1^)	300	1575	662	381	58
	CD	0.46	0.90	0.79	0.10	12.17
	DH (m)	12.43	22.98	17.51	1.90	10.88
	DD (cm)	16.88	34.55	25.48	5.32	20.89

Note: Area: sample plot area, CR: crown ratio, HCB: trunk height to crown base, D: diameter at breast height, H: tree height, SD: stand density, CD: canopy density, DH: dominant tree height, DD: dominant tree diameter.

### Modeling approach

#### Two-level NLME models

Tree CR mixed-effect models with two-level random effects were formulated according to multilevel nonlinear mixed-effect model techniques in this study [34]. That is, blocks were used as the first level random effect and sample plots were nested in each of block, showing interaction, as the second level random effect:

$$CR_{ijk}=f(\phi_{ijk},t_{ijk})+\varepsilon_{ijk},i=1,\ldots ,M,j=1,\ldots ,M_{i},k=1,\ldots ,n_{ij}$$ (1)

where *CR*_*ijk*_ is the crown ratio of the *k* ^*th*^ tree on the *j* ^*th*^ plot nested within the *i* ^*th*^ block, *M* is the number of blocks, *M*_*i*_ is the number of plots within the *i* ^*th*^ block, *n*_*ij*_ is the number of observations in the *j* ^*th*^ plot of the *i* ^*th*^ block, and *f*(·) is a real-valued and differentiable function of a group-specific parameter vector ***ϕ***_*ijk*_ and a covariate vector **t**_*ijk*_. The within-group error *ε*_*ijk*_ that accounts for within-group variance and correlation was assumed to follow a normal distribution that has an expectation of zero and a positive-definite variance-covariance structure **R**_*ij*_ [35]. **R**_*ij*_ is generally expressed as a function of the parameter vector λ [24]:

$$\varepsilon_{ijk}\sim N(0,R_{ij}(\lambda ))$$

Moreover, ***ϕ***_*ijk*_ can be expressed as [16]:

$$\phi_{ijk}=A_{ijk}\beta +B_{i,jk}u_{i}+M_{ijk}u_{ij},u_{i}\sim N(0,\psi_{1}),u_{ij}\sim N(0,\psi_{2})$$ (2)

where **β** is a *p*-dimensional vector of fixed effects, meaning the first-level random effects, **u**_*i*_ is independently normal distributed *q*_1_-dimensional vector with zero means and variance- covariance matrix **Ψ**_1_, indicating the second-level random effects, **u**_*ij*_ is independently normal distributed *q*_2_-dimensional vector with zero means and variance-covariance matrix **Ψ**_2_, and **A**_*ijk*_, **B**_*ijk*_ and **M**_*ijk*_ are design matrices, and **u**_*i*_, **u**_*ij*_ and *ε*_*ijk*_ are independent of each other.

#### Predictor variables

Growth of individual trees is potentially affected by three groups of variables: tree size and vigor effects, site condition effects and competition [36–39]. In this study, a total of 14 variables, including 2 tree size and vigor effects related variables, 1 site condition effects related variable, and 11 competition effects related variables (Table 4), were used to evaluate their effects on tree CR.

**Table 4:** A total of 14 candidate variables used for developing crown ratio model.

Groups of variables	Variables
Tree size and vigor effects	diameter at breast height (D), total tree height(H)
Site condition effects	Meyer’ site index
Competition effects	stand density (SD), canopy density (CD), dominant tree height (DH), plot arithmetic mean diameter (AMD), plot dominant tree diameter (DD), plot quadratic mean diameter (QMD), number of trees with diameter larger than the target tree (LDN), total diameter of all trees with diameter larger than the target tree (LDTD), mean diameter of all trees with diameter larger than the target tree (LDMD), total basal area of all trees with diameter larger than the target tree (LDTBA), and mean basal area of all trees with diameter larger than the target tree (LDMBA)

The tree and stand variables were selected using a stepwise regression procedure. The measurements of the variables were first analyzed by graphical examination and correlation statistics [36]. Different combinations of the variables and their logarithmic transformations in linear models were then tested based on the coefficient of determination *R* ^*2*^. All the calculations were performed using R/S-Plus *nls* function [40].

#### Model selection

The base model used to develop the NLME model of tree CR was determined from a total of 10 candidate models (Table 1) with selected predictors based on the performance of model fitting and prediction. The models were first fit to the data from 2166 trees of 74 PSPs and their predictions were then compared with the observations from 968 trees of 38 PSPs for the validation dataset. Nonlinear regressions were carried out using ordinary nonlinear least square (ONLS) technique with the R/S-Plus *nls* function [40]. The following four statistical criteria were used to select the model that had the highest accuracy for both fitting and prediction [25, 41]:

$$\bar{e}=\sum e_{i}/N=\sum (CR_{i}-\overset{\wedge }{CR_{i}})/N$$ (3)

$$\xi =\sum {\left(e_{i}-\bar{e}\right)}^{2}/(N-1)$$ (4)

$$\delta =\sqrt{{\bar{e}}^{2}+\xi }$$ (5)

$$R^{2}=1-\sum {\left(CR_{i}-\overset{\wedge }{CR}\right)}^{2}/\sum {\left(CR_{i}-\bar{CR}\right)}^{2}$$ (6)

where *CR*_*i*_ and $\overset{\wedge }{CR_{i}}$ are the observed and predicted values of tree CR for the *i* ^*th*^ observation (*i* = 1,…,*N*), *N* is the total number of observations, $\bar{CR}$ is the mean of the observed tree CR values, $\bar{e}$ is the mean prediction error, *ξ* is the variance of prediction errors, *δ* is the root mean square error, *R* ^2^ is the coefficient of determination. *δ* combines the mean prediction error ($\bar{e}$) and the variance of prediction errors (*ξ*), giving a robust measure of the overall model accuracy, and therefore was selected as a primary criterion for model evaluation [25]. The selected model with predictor variables was then used as a base model to construct NLME model of tree CR.

#### Construction of parameter effects

Several approaches were proposed to determine which parameters should be modeled as mixed effects in the base model [21, 42, 43]. In this study, we fitted all possible combinations of random effects on parameters including intercept and slope terms in the base model and selected the one with the smallest Akaike’s information criterion (AIC) and the largest log-likelihood (Loglik) [34]. To avoid over parameterization, the likelihood ratio test (LRT) was also used [16, 43].

#### Determining Ψ_1_ and Ψ_2_ structure

The variance-covariance matrices for the random effects, **Ψ**_**1**_ and **Ψ**_**2**_, define the variability that exists among the sample plots and blocks, respectively. As in the study of Calama and Montero [44], both **Ψ**_**1**_ and **Ψ**_**2**_ are assumed unstructured in this study.

#### Determining R structure

To account for the within-plot heteroscedasticity and autocorrelation in **R**_*ij*_ [24, 35], we applied the approach suggested by Davidian and Giltinan [35]:

$$R_{ij}=\sigma^{2}G_{ij}^{0.5}\Gamma_{ij}G_{ij}^{0.5}$$ (7)

where *σ* ^2^ is a scaling factor for error dispersion [21], given by the value of residual variance of the estimated model, **G**_*ij*_ is a *n*_*ij*_ × *n*_*ij*_ diagonal matrix explaining the variance of within-plot heteroscedasticity and **Γ**_*ij*_ is a *n*_*ij*_ × *n*_*ij*_ matrix accounting for the within-plot autocorrelation structure of errors.

Three widely used variance models, including exponential model Eq (8), power model Eq (9) and constant plus power model Eq (10) [16] each with different predictors, were used to account for heterogeneity in variance. The LRT and AIC were employed to determine an appropriate variance model.

$$\text{var}(\varepsilon )=\sigma^{2}\text{exp}(2\gamma x)+\tilde{\varepsilon }$$ (8)

$$\text{var}(\varepsilon )=\sigma^{2}x^{2\gamma }+\tilde{\varepsilon }$$ (9)

$$\text{var}(\varepsilon )=\sigma^{2}{\left(\gamma_{1}+x^{\gamma_{2}}\right)}^{2}+\tilde{\varepsilon }$$ (10)

where *x* is one of the selected predictors; *γ*, *γ*_1_ and *γ*_2_ are the estimated parameters; $\tilde{\varepsilon }$ is an error term. In addition, two autocorrelation structures, AR (1) (autoregressive process of order one) and ARMA (autoregressive moving average process) for matrix **Γ**_***i***_, were evaluated, and the one that had a higher fitting accuracy (i.e., smaller AIC) and provided the expected residual pattern was selected.

#### Parameter estimation

The parameters in Eq (1) were estimated by maximum likelihood (ML) estimation method using the Lindstrom and Bates (LB) [19] algorithm which alternates between two steps: a penalized nonlinear least square (PNLS) step and a linear mixed-effect (LME) step [16, 19]. This algorithm was implemented using the R/S-Plus *nlme* function [16] and for its details, readers can refer to Lindstrom and Bates [19] and Pinheiro and Bates [16].

#### Model prediction and evaluation

When two-level NLME models are used to predict tree CR, both population average (PA) and subject-specific (SS) responses [15, 34, 45] are often considered. The former is related to the fixed effect response and prediction of tree CR in the stands where independent stand or tree variables required by the models are measured, but tree CR measurements are not collected. The latter is related to the predictions of tree CR in the stands where in addition to the independent variables, the dependent variable tree CR is also measured in a sub-sample of trees. In order to reduce both measurement cost and potential errors, as Calama et al [15] and Temesgen et al [46] suggested, four randomly selected trees within each plot were measured for estimation of the random effect parameters in this study [15, 46].

#### Population average model

A population average (PA) model means that it contains the fixed effects as global parameters and the random effects as zero and has the following general form [34]:

$$\overset{\wedge }{CR_{ijk}}=f({\hat{\phi }}_{ij},t_{ijk})+\varepsilon_{ijk}$$ (11)

Where $\overset{\wedge }{CR_{ijk}}$ are the predicted values of tree CR for the *k* ^th^ tree in the *j* ^th^ plot in the *i* ^th^ block, **t**_*ijk*_ are other stand and tree variables for the corresponding tree, and ${\hat{\phi }}_{ij}$ is the estimates of PA model parameters and contains $\hat{\beta }$.

#### Subject-specific response at block level

When a model accounts for the effects of block on tree CR and ignores other effects such as the interaction of blocks and sample plots, its parameter estimates ${\hat{\phi }}_{ij}$ in Eq (11) contain both $\hat{\beta }$ and ${\hat{u}}_{i}(i=1,\ldots ,M)$. Usually, the random effect parameter estimates ${\hat{u}}_{i}$ are obtained by an empirical best linear unbiased prediction (EBLUP) approach [20, 25, 34].

$${\hat{b}}_{i}\approx \hat{G}{\hat{C}}_{i}^{T}{\left({\hat{R}}_{i}+{\hat{C}}_{i}\hat{G}C_{i}^{T}\right)}^{-1}{\hat{e}}_{i}$$ (12)

where the superscript *T* denotes the matrix transpose operation, ${\hat{b}}_{i}={\hat{u}}_{i}$ is the *q*_1_ × 1 dimensional random effects from blocks, $\hat{G}={\hat{\psi }}_{1}$ is the *q*_1_ × *q*_1_ dimensional variance-covariance matrix, ${\hat{C}}_{i}={\hat{Z}}_{i}$ is the *n*_i_ × *q*_1_ dimensional design matrix of the partial derivatives of the nonlinear function *f*(·) with respect to random effect parameters **u**_*i*_, $n_{i}=\sum_{i=1}^{M_{i}}n_{ij}$. The values of blocks in the data used for both model fitting and validation were identical. Thus, the values of ${\hat{u}}_{i}$ do not need to be recalculated in prediction. That is, they would be equal to the estimates obtained when the model is fitted.

### Subject-specific response at both plot and block levels

In addition to $\hat{\beta }$, ${\hat{\phi }}_{ij}$ in Eq (11) also contains both ${\hat{u}}_{i}$ and ${\hat{u}}_{ij}$. That is, it simultaneously incorporates the block effects and the interaction of blocks and sample plots. The random effect parameters were calculated by Eq (12). The specific structure of ${\hat{b}}_{i}$,$\hat{G}$ and ${\hat{C}}_{i}$ (*i* = 1,…,*M*) in the Eq (12) in this case are presented in Appendix 1.

### Model assessment

Statistics $\bar{e}$, *ξ*, *δ* and *R* ^2^ calculated by Eqs (3)–(6), and LRT were applied to assess the predictive ability of the developed tree CR models using both fitting and validation datasets. The obtained mixed-effect models were compared based on Loglik, AIC and LRT.

## Results

### Selection of base model

To avoid over-parameterization and collinearity in the models, only those variables that had statistically significant contributions to improving the quality of the models fit to the data were selected. The obtained variables were: D, DH and plot dominant tree diameter (DD). Especially, the variables D and DH were strongly correlated with tree CR and included in most of the candidate models in Table 1. All the candidate models with selected predictors converged except II.9 and II.10 in Table 1. The statistics for accuracy measures Eqs (3)–(6) to assess the performance of converged candidate models for both fitting and validation are calculated (Table B in S1 File). It is found that the values of the same statistics were very similar to each other among the models. For the data used to fit the models, the values of $\bar{e}$ for all the candidate model were very close to 0, and the values of both *ξ* and *δ* had the same range of 0.1681 to 0.1687. The coefficients of determination varying from 0.2469 to 0.2554 were very small. For the data used to validate the models, the values of $\bar{e}$, *ξ* and *δ* for all the models had small ranges of -0.0174 to -0.0147, 0.1712 to 0.1717, and 0.1718 to 0.1725, respectively. But, the model validation results showed that model II.1 had the smallest values of mean prediction error, $\bar{e}=-0.0147$, variance of prediction error, *ξ* = 0.1712, and root mean square error, *δ* = 0.1718, suggesting a slightly higher prediction accuracy than the others. Additionally, another advantage for model II.1 is that its predictions are constrained to be between 0 and 1, i.e., 0% to 100% CR. Thus, the base model selected to construct the CR mixed-effect model is:

CRijk=1[1+exp(ϕ0+ϕ1Dijk+ϕ2DHij+ϕ3DDij)]+εijk (13)

where *D*_*ijk*_ are the diameter at breast height (cm) of the *k* ^th^ tree in the *j* ^th^ plot in the *i* ^th^ block stand density index class, *DH*_*ij*_ and *DD*_*ij*_ are the plot dominant tree height (m) and plot dominant tree diameter at breast height (cm), respectively, of the *j* ^th^ plot of the *i* ^th^ block stand density index class, and *ϕ*_0_, *ϕ*_1_, *ϕ*_2_ and *ϕ*_3_ are the model parameters and other variables as defined previously.

### Mixed-effect model

Considering four model parameters (*ϕ*_0_-*ϕ*_3_) involved and both block effects and interaction of blocks and sample plots, a total of 15 different combinations of random effects were obtained for Eq (13). The mixed-effect model with each of the combinations was fitted to the data. It was found that only 10 mixed-effect model alternatives converged (Table C in S1 File). The following model Eq (14) with block effects and the interaction showed the smallest AIC, -1919.58, and the largest Loglik, 970.79 (Table C in S1 File):

$$CR_{ijk}=1/\left[1+\text{exp}(\beta_{0}+u_{0i}+u_{0ij}+(\beta_{1}+u_{1i}+u_{1ij})D_{ijk}+\beta_{2}DH_{ijk}+\beta_{3}DD_{ij})\right]+\varepsilon_{ijk}$$ (14)

where *β*_0_, *β*_1_, *β*_2_ and *β*_3_ are fixed-effect parameters, *u*_1i_ and *u*_3i_ are random-effect parameters caused by blocks on *ϕ*_1_ and *ϕ*_3_, respectively, *u*_1ij_ and *u*_3ij_ are random-effect parameters caused by the interaction of blocks and sample plots on *ϕ*_1_ and *ϕ*_3_, respectively.

**Fig 2:**
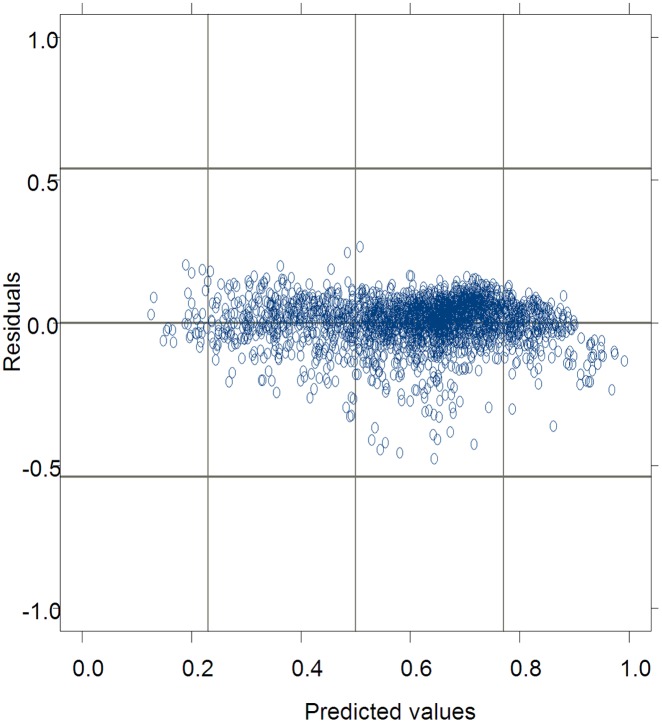
The residuals of predicted crown ratio values from Eq (14) graphed against the predicted values for Mongolian oak in Wangqing Forest Bureau of northeast China.

The residuals from Eq (14) were graphed against the predicted values for the data used for model fitting in Fig 2. The absolute values of the residuals tended to decrease as the observed tree CR values increased. It indicated that some heteroscedasticity remained even after incorporating the effects of blocks and the interaction effects of blocks and sample plots in the nonlinear mixed-effects CR model (14). Furthermore, the empirical autocorrelation function (ACF) for Eq (14) indicated that the autocorrelation was significant at a risk level of 0.05 among residuals within plots.

### Within-plot variance-covariance (R) structure

The assessment statistics based on three variance models with each selected predictor (D, DH, and DD) for Eq (14) were shown in Table 5. For comparison purpose, the assessment results of this model were also derived and given when the variances of the error term *ε*_*ijk*_ was assumed homogeneous. Power model Eq (8) with DH as a predictor failed to converge. In the case of homogeneous variance, whether D or DD or DH was used as a predictor, the values of AIC and Loglik were significantly different from those using power model Eq (8), exponential model Eq (9) and constant plus power model Eq (10) at a risk level of 0.05. Even with random effects in the parameters, heteroscedasticity still existed in the mixed-effect model Eq (14) for tree CR. Among these variance models, exponential model Eq (9) with DH as a predictor showed the highest accuracy for model fitting (Table 5) (AIC = -1987, Loglik = 1006). Eq (14) with the Autocorrelation structure AR (1) produced a smaller AIC (AIC = -1950) than that with the ARMA (1, 1) (AIC = -1931). Therefore, Eq (14) was fitted with an AR (1) autocorrelation structure and an exponential variance model. The final nonlinear mixed-effects CR model was:

$$\left\{ \begin{array}{l} CR_{ijk}=1/\left[1+\text{exp}(\beta_{0}+u_{0i}+u_{0ij}+(\beta_{1}+u_{1i}+u_{1ij})D_{ijk}+\beta_{2}DH_{ijk}+\beta_{3}DD_{ij})\right]+\varepsilon_{ijk}\\ \varepsilon_{ij}={\left(\varepsilon_{ij1},\ldots ,\varepsilon_{ijn_{ij}}\right)}^{T}\sim N\left(0,R_{ij}=\sigma^{2}G_{ij}^{0.5}\Gamma_{ij}G_{ij}^{0.5}\right)\\ G_{ij}^{}=diag\left(\underset{n_{ij}}{\underset{︸}{\sigma^{2}exp(2\gamma DH_{ij}),\ldots ,\sigma^{2}exp(2\gamma DH_{ij})}}\right)\\ \Gamma_{ij}=\frac{\sigma^{2}}{1-\rho^{2}}\left(\begin{array}{cccc} 1 & \rho & \cdots & \rho^{\left(n_{ij}-1\right)}\\ \rho & 1 & \cdots & \rho^{\left(n_{ij}-2\right)}\\ \vdots & \vdots & \ddots & \vdots \\ \rho^{\left(n_{ij}-1\right)} & \rho^{\left(n_{ij}-2\right)} & \cdots & 1 \end{array}\right) \end{array}\right.,$$ (15)

where *ρ* is an estimated parameter in the matrix **Γ**_ij_ with the autocorrelation structure AR (1), and other variables and coefficients in this model defined as above.

**Table 5:** Performance assessment of mixed-effect model Eq (14) using measurements of crown ratio with different variance models.

Variance	D				DD				DH			
model	AIC	Loglik	LR	p value	AIC	Loglik	LR	p value	AIC	Loglik	LR	p value
1	-1920	971			-1920	971			-1920	971		
PF	-1965	994	47.26	<0.0001	-1965	994	46.95	<0.0001	None		F	F
EF	-1970	997	52.02	<0.0001	-1957	991	39.72	<0.0001	-1987	1006	70.03	< 0.0001
CPF	-1963	994	47.25	<0.0001	-1963	994	46.97	<0.0001	-1962	994	46.97	< 0.0001

Note: D: diameter at breast height, DD: dominant tree diameter, DH: dominant tree height, AIC: Akaike information criterion, Loglik: log-likelihood, LR: likelihood ratio, Variance model 1 means that the variances are homogeneous, PF: power model—Eq (8), EF: exponential model—Eq (9), CPF: constant plus power model—Eq (10), F denotes a model failing to converge.

### Parameter estimation

Table 6 shows the parameter estimates and performance results for fixed effects and variance-covariance for Eqs (13), (14) and (15). Eq (13) had only one variance parameter (*σ* ^2^) because its error variance was assumed to be homogeneous. Eqs (14) and (15) had much smaller AIC values and larger Loglik values than Eq (13), which implied that the effects of blocks and the interactions of blocks and sample plots on tree CR were significant. Among these models, Eq (15) had the smallest AIC, -1993, and the largest log-likelihood, 1009. After the parameter estimates were put into Eq (15), the tree CR model for Mongolian oak in northeast China is:

$$\overset{\wedge }{CR_{ijk}}=1/[1+\text{exp}(0.3990+u_{0i}+u_{0ij}+(-0.0168+u_{1i}+u_{1ij})D_{ijk}+\;0.0007DH_{ij}-0.0361DD_{ijk})]+\varepsilon_{ijk}$$ (16)

Where

$$u_{i}=\left[\begin{array}{l} u_{0i}\\ u_{1i} \end{array}\right]\sim N\left\{\left[\begin{array}{l} 0\\ 0 \end{array}\right],{\hat{\psi }}_{1}=\left(\begin{array}{cc} 0.1643 & 0.1730\\ 0.1730 & 0.0070 \end{array}\right)\right\},$$

$$u_{ij}=\left[\begin{array}{l} u_{0ij}\\ u_{1ij} \end{array}\right]\sim N\left\{\left[\begin{array}{l} 0\\ 0 \end{array}\right],{\hat{\psi }}_{2}=\left(\begin{array}{cc} 0.2058 & -1\\ -1 & 0.0042 \end{array}\right)\right\},$$

$$\varepsilon_{ij}\sim N\left(0,{\hat{R}}_{ij}=0.3622{\hat{G}}_{ij}^{0.5}{\hat{\Gamma }}_{ij}{\hat{G}}_{ij}^{0.5}\right),$$

$${\hat{G}}_{ij}^{}=diag\left(\underset{n_{ij}}{\underset{︸}{0.3622\text{exp}(-0.1042DH_{ij}),\ldots ,0.3622\text{exp}(-0.1042DH_{ij})}}\right)$$

$${\hat{\Gamma }}_{ij}=0.3636\left(\begin{array}{cccc} 1 & 0.0618 & \cdots & 0.0618^{\left(n_{ij}-1\right)}\\ 0.0618 & 1 & \cdots & 0.0618^{\left(n_{ij}-2\right)}\\ \vdots & \vdots & \ddots & \vdots \\ 0.0618^{\left(n_{ij}-1\right)} & 0.0618^{\left(n_{ij}-2\right)} & \cdots & 1 \end{array}\right)$$

**Table 6:** Parameter estimates and performance assessment results of models.

	Parameter estimates	Eq (13)	Eq (14)	Eq (15)	Eq (17)
Fixed-effect	${\hat{\beta }}_{0}$	0.6827	0.3650	0.3990	0.6026
parameters	${\hat{\beta }}_{1}$	-0.0126	-0.0166	-0.0168	-0.0169
	${\hat{\beta }}_{2}$	-0.0097	-0.0006	0.0007	-0.0046
	${\hat{\beta }}_{3}$	-0.0413	-0.0325	-0.0361	-0.0386
Canopy density	${\hat{\beta }}_{0}^{\left(1\right)}$	—	—	—	-0.1054
effects	${\hat{\beta }}_{0}^{\left(2\right)}$	—	—	—	0.0199
	${\hat{\beta }}_{0}^{\left(3\right)}$	—	—	—	-0.0662
	${\hat{\beta }}_{0}^{\left(4\right)}$	—	—	—	0.0344
Variance	${\hat{\sigma }}_{Block0}^{2}$	—	0.1627	0.1643	0.1733
parameters	${\hat{\sigma }}_{block1}^{2}$	—	0.0071	0.0070	0.0067
	${\hat{\rho }}_{Block01}$	—	0.324	0.173	0.185
	${\hat{\sigma }}_{Block*Plot0}^{2}$	—	0.2251	0.2058	0.2086
	${\hat{\sigma }}_{block*Plot1}^{2}$	—	0.0073	0.0042	0.0049
	${\hat{\rho }}_{Block*Plot01}$	—	-0.94	-1	-1
	${\hat{\sigma }}^{2}$	0.1683	0.1514	0.3622	0.3620
	$\hat{\gamma }$	—	—	-0.0521	-0.0521
	$\hat{\rho }$	—	—	0.0618	0.0613
Model	AIC	-1568	-1920	-1993	-2063
assessment	Loglik	789	971	1009	1045

Note: AIC, Akaike information criterion; Loglik, log-likelihood; $\hat{\rho }$, parameter estimate for autoregressive process of order one.

### Model prediction

Table 7 presents the statistics of model performance based on the data used for both model fitting and validation for the base model Eq (13), and two mixed-effect models Eqs (14) and (15) in three cases: PA, block, and block plus interaction effects of blocks and sample plots. In Table 7, Eq (13) itself was regarded as a population average (PA) model and its parameters were directly estimated by fitting to the data without random effects, while Eq (14) PA model only consisting of the components that account for the fixed effects was indirectly obtained by first fitting the model that contained both fixed effects and random effects to the data and then removing the components related to the random effects. This was also applied to Eq (15) PA model. The results showed that although all the models produced the mean prediction errors that were not significantly different from zero at a significance level of 0.05, the prediction accuracy of the models with both the block effects and interaction was the highest, followed by the models with block effects alone, and by the PA models. The major difference between Eqs (14) and (15) was seen in the case of considering both the block effects and interaction. Eq (15) had much smaller statistics of $\bar{e}$, *ξ* and *δ* than Eq (14) although both models slightly under-predicted tree CR and resulted in the mean prediction errors that were not significantly different from zero at a risk level of 0.05.

**Table 7:** Performance assessment results of the models.

Model No.	Model fitting				Model validation		
	$\bar{e}$	ξ	δ	R^2^	$\bar{e}$	ξ	δ
Eq (13)	0	0.1681	0.1681	0.2554	-0.0147	0.1712	0.1718
Eq (14)							
PA	-0.0016	0.1686	0.1686	0.2444	-0.0162	0.1702	0.1709
B	0.0044	0.1547	0.1547	0.4579	-0.0095	0.1647	0.1650
B+B*Plot	0.0017	0.0861	0.0861	0.6359	0.0034	0.1143	0.1144
Eq (15)							
PA	-0.0012	0.1687	0.1687	0.2439	-0.0159	0.1704	0.1712
B	0.0038	0.1549	0.1549	0.4568	-0.0094	0.1648	0.1650
B+B*Plot	0.0012	0.0814	0.0814	0.6435	0.0025	0.1054	0.1054
Eq (17)	0.0005	0.0412	0.0412	0.6827	0.0012	0.0653	0.0653

Note: $\bar{e}$: mean prediction error, *ξ*: variance of prediction error, *δ*: root mean square error, R^2^: coefficient of determination between the observed and predicted values, B: block, and B*Plot: interaction of block with plot.

Compared to that from Eq (13), the prediction accuracies of Eqs (14) and (15) with both the block effects and the interaction of blocks and sample plots were much higher. Without the interaction effects, moreover, Eqs (14) and (15) led to very small coefficients of determination (R^2^ varying from 0.2439 to 0.4579). Adding the interaction effects into both models increased the coefficients to 0.6359 and 0.6435 for Eqs (14) and (15), respectively. Based on the results of model validation, Eq (15) with the interaction effects decreased the root mean square error by 38.65% and 38.33% compared to Eqs (13) and (14) (PA model). These implied that the random effects from the interaction of blocks and sample plots on tree CR were substantially large.

**Fig 3:**
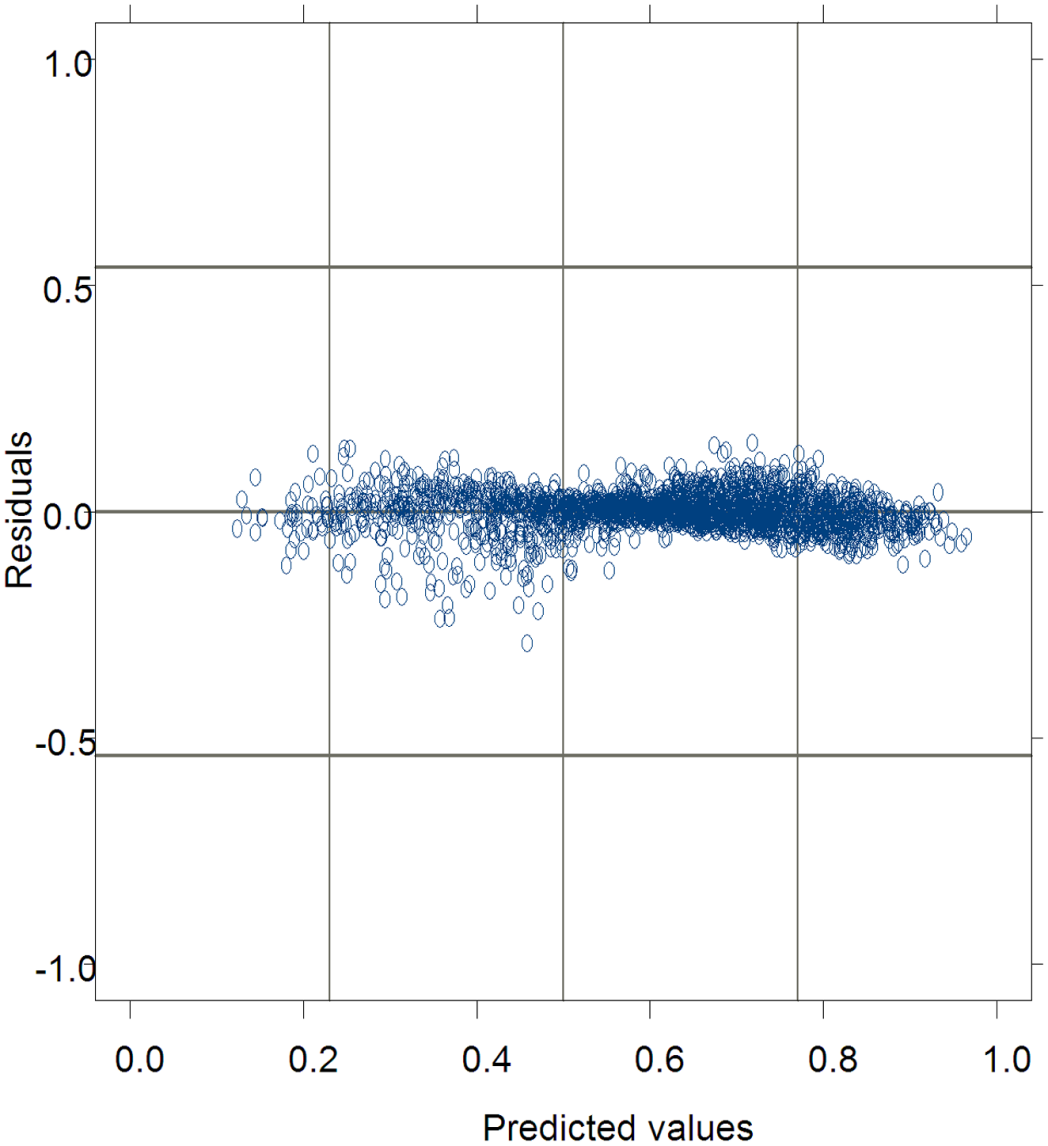
The residuals of predicted crown ratio values from Eq (15) graphed against the predicted values for Mongolian oak in Wangqing Forest Bureau of northeast China.

The residuals from Eq (15) with the interaction effects of blocks and sample plots were graphed against the predicted values for the data used for model fitting in Fig 3. Compared to those in Fig 2, the residuals from Eq (15) with the interaction effects, especially the under-predictions, were greatly decreased. This further indicated that the exponential variance model with HD as a predictor accounted for heteroscedasticity effectively. Similar results of the residuals from the Eq (15) were also found for the data used for model validation. Additionally, the error structures of Eq (15) did not show any signs of autocorrelation. Therefore, Eq (15) was promising for predicting tree CR of Mongolian oak.

### Model extension

Our preliminary analyses revealed that the correlations between tree CR and the predictors varied with stand density class. This implied that the accuracy of predicting tree CR could be potentially increased by taking into account the stand density classes. To account for the variation among stand density classes, dummy variables (*K*_1_, *K*_2_, *K*_3_ and *K*_4_) with values of 1 and 0 were introduced into the model. That is, *K*_1_ = 1, *K*_2_ = 1, *K*_3_ = 1, and *K*_4_ = 1 meant the first, second, third and fourth stand density class, respectively, and *K*_1_ = *K*_2_ = *K*_3_ = *K*_4_ = 0 implied the fifth stand density class. Adding the dummy variables into Eq (15) led to following model:

$$CR_{ijk}=1/[1+\text{exp}(\beta_{0}+u_{0i}+u_{0ij}+\beta_{0}^{\left(1\right)}K_{1}+\beta_{0}^{\left(2\right)}K_{2}+\beta_{0}^{\left(3\right)}K_{3}+\beta_{0}^{\left(4\right)}K_{4}+(\beta_{1}+u_{1i}+u_{1ij})D_{ijk}+\;\beta_{2}DH_{ij}+\beta_{3}DD_{ij}]+\varepsilon_{ijk}.$$ (17)

**Fig 4:**
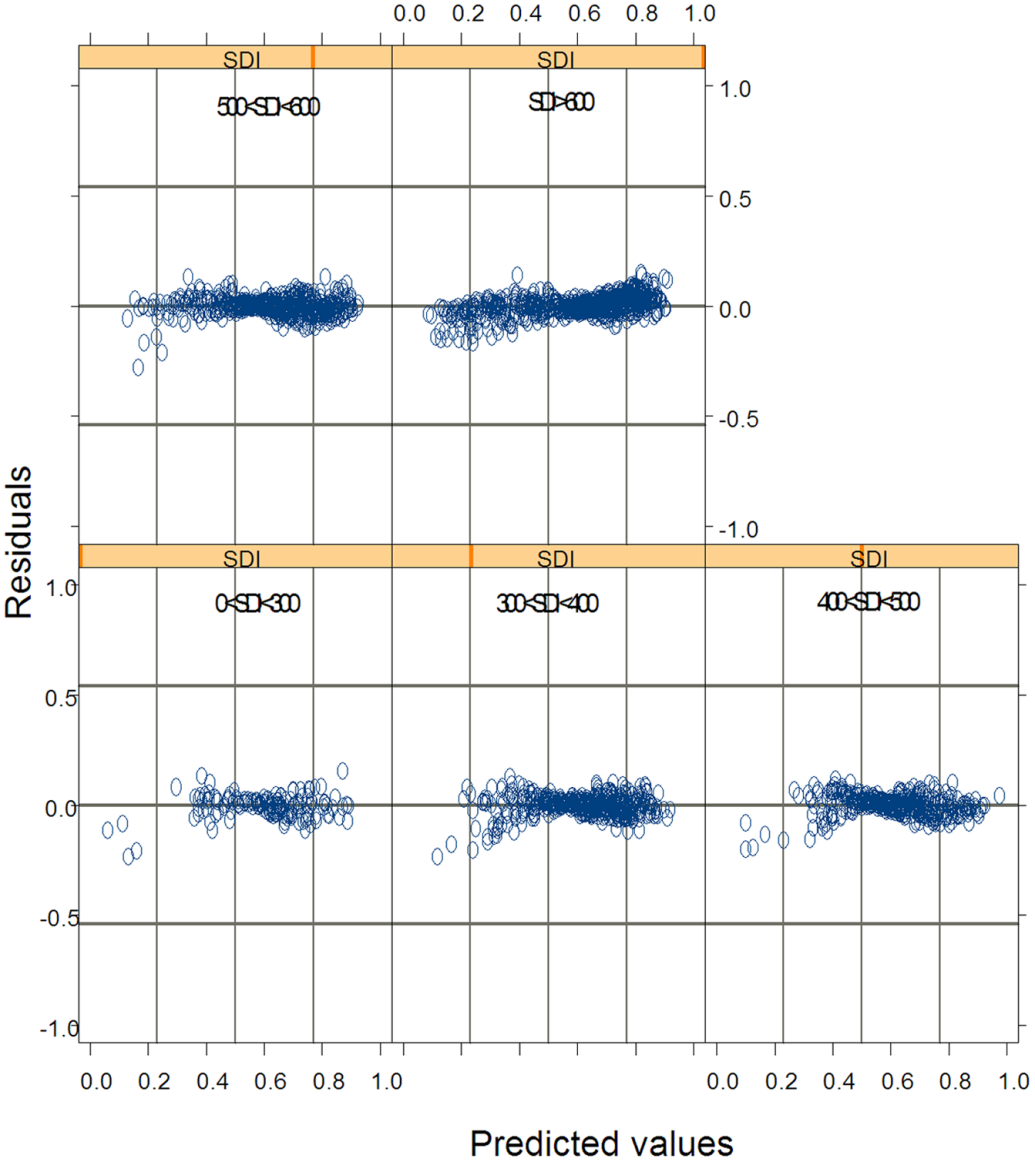
Residuals of predicted crown ratio values from Eq (17) for each of five stand density classes graphed against the predicted values for Mongolian oak in Wangqing Forest Bureau of northeast China.

The estimates of model parameters for Eq (17) were listed in Table 6 and its statistics of performance to predict tree CR were shown in Table 7. In both tables, Eq (17) was compared with Eqs (13)–(15) based on the values of the performance statistics. The results showed that compared to Eqs (15) and (17) decreased the value of AIC by 3.51% and increased the value of Loglik by 3.57% (Table 6). Eq (17) also increased the coefficient of determination by 167.31%, 7.36%, and 6.09% compared to those from Eqs (13), (14) and (15), respectively. Based on the results from the validation data, Eq (17) led to a root mean square error of 0.0653, decreasing by 61.99%, 42.92%, and 38.05% compared to those from Eqs (13), (14) and (15). The residuals from Eq (17) were graphed against the predicted values of tree CR for five stand density classes (Fig 4). The results implied that if the stand density of each sample plot was known, Eq (17) in which stand density class was introduced through dummy variables can result in further improvement of predictions.

## Discussion and Conclusions

A nonlinear mixed-effects model was appealing for the analysis of correlated hierarchical structure data because of its flexibility to account for the covariance structures that are not taken into account in traditional regression approaches. A modified logistic model (Eq (15)) with nonlinear mixed-effects model approach at both block and plot levels was recommended for modelling CR of Mongolian oak trees. The results (Tables 6 and 7) showed that a random effects model provided higher accuracy of prediction for the datasets used for both model fitting and validation compared to a similar model without random effects. It is also worth pointing out that the variance-covariance matrices for the random effects and within-plot errors play a more important role in prediction. If we ignore the random effects, within-plot heterogenity and correlation, and apply the ordinary least square approach to the final chosen model (Eq (15)), the statistics ($\bar{e}$, *ξ* and *δ*) for the prediction CR are obviously much larger than those obtained by appropriate variance-covariance structure (see Table 7).

In this study, three tree and stand variables, including D, DD and DH, were selected and involved in the mixed-effect model of tree CR. This finding was supported by other tree CR model studies in which D was often used an independent variable [1, 2, 10, 11]. As Hasenauer and Monserud [5] and Soares and Tomé [1] concluded, the parameter *β*_1_ in our Eq (15) was negative, meaning that increasing D would result in a larger value of tree CR (Table 6). This implied that the effect of D on tree CR was significant. In fact, D is one of the most important tree variables and usually applied to account for stand structure, tree vigor and competition capacity. Soares and Tomé [1] also included DH in their tree CR model for *Eucalyptus globules* Labill plantations. In most forest growth studies, DH often implies development of trees and stands [47]. On the other hand, DH is a measureable stand characteristic and indicates the site quality in terms of stand growth and yield [48]. In the study of Soares and Tomé [1], greater values for DH resulted in smaller values of CR for *Eucalyptus globulus* Labill in the north and central coastal regions of Portugal. The finding in this study was consistent with this conclusion, that is, tree CR decreased as DH increased because the parameter *β*_2_ in the model (15) was positive. In addition, the effect of dominant tree diameter DD on CR was also found to be significant in this study and the negative *β*_3_ implied that tree CR increased as DD increased.

Monserud and Sterba [49], Temesgen et al. [10], and Leites et al. [2] found that many other stand or tree variables had significant effects on tree CR, such as tree height (H), and H/D, named as tree slenderness coefficient [3]. In this study, these variables were also examined and it was found that their contributions to the prediction accuracy of the tree CR model were significant. However, measuring H of each tree is prohibitively high cost and time-consuming [15, 47]. Therefore, H and H/D were not selected as predictors in the model (15) to enhance the feasibility and practicality of the model, Site index (SI) affects tree growth significantly ([34, 36, 45, 50], but the effects of Meyer’ SI on CR is insignificant in this study. This is mainly because (i) DH contained in the proposed model could reflect the site quality effectively; (ii) in Wangqing Forest Bureau of northeast China, the environmental and climatic factors greatly vary from sample plot to sample plot due to the complication of environmental and climatic conditions so that the relationship between tree CR and SI greatly differs from place to place; and (iii) introducing SI would complicate the model for a very small portion of gain in predictability. Thus, the stand and tree variables such as SI were not selected.

The tree CR is widely used as a predictor variable to predict growth and yield of trees and forests and also used as a key factor to determine target trees in the management of close-to-nature forests [51–53]. In practice, forest inventory data do not necessarily include the measurements of CR. The mixed-effect CR model (such as Eq (15)) proposed in this study can be used to predict tree CR in those cases. The method that is used to predict CR based on the mixed-effect CR model in practical application consists of two steps: parameter estimation and prediction, which is similar as the approach that proposed in this study. For different tree species or the same tree species but it comes from different area, the values of parameters in the mixed-effect CR model may be different. Therefore, the parameters in the model should be estimated before prediction. In addition, it is also noted that when predicting tree CR using the mixed-effect models such as Eq (15), the random-effect parameters should be estimated from the prior information obtained from the observations of the dependent variable [15, 25, 43, 44, 54]. In addition to the predictors D, DD and DH, tree CR has to be measured from a small sub-sample of trees so that the random-effect parameters can be estimated using EBLUP theory [20, 25, 34]. The appropriate number of trees to be measured for estimating the random-effect parameters has been discussed in the literature [34, 44, 46, 47]. Temesgen et al. [46] and Yang et al. [34] suggested that the more trees used for estimation of random-effect parameters, the higher the prediction accuracy. However, as the number of trees increases, the decrease of prediction error will become insignificant, while the cost of inventory will greatly increase. In fact, the analysis of data using 1–6 trees revealed the accuracy of prediction with four trees was similar to that with five or six trees. As Calama and Montero [44] suggested, therefore, in this study four randomly selected trees were measured for estimation of random-effect parameters.

The prediction of tree CR would have some potential random error. The value of the error varies mainly depending on the prediction accuracy of the mixed-effect CR model (such as (15)). Therefore, the predictions from the forest growth and yield models that use predicted tree CR as a predictor are associated with uncertainty and subject to the random error in the predicted tree CR. One direct method to reduce the uncertainty is using an accurate CR model to predict CR. This is why we recommended the use of the nonlinear mixed-effect modelling approach to develop a CR model for improving the prediction accuracy in this study. In addition, several other approaches, such as simultaneous equations [55], errors-in-variables (EIV) models [56] and Bayesian estimation [57], could also be used to reduce the uncertainty in the case of using the predicted CR as a predictor variable in the forest growth and yield modelling.

Measuring tree CR is subject to error, even though stand and tree attributes are commonly assumed to be measured without error [58, 59]. The measurement errors due to mistakes of field crew and faulty instrument can be substantial [58, 60]. For example, tree CR is generally measured with standard height measurement instrument, but, when the crown is uneven, one often visually rearranges the crown branches to obtain the values of tree CR. There is ample evidence in the literature about the ambiguity of visual estimation of tree characteristics. The studies of Nicholas et al. [61] and Ghosh et al. [62] highlighted the degree of variation that can arise from subjective measurements of tree and stand variables. All the existing tree CR models (Table 1) including the models developed in this study are assumed that (i) tree CR is a random variable; and (ii) the independent variables are fixed and observed without error. It is well known that the violation of the second assumption may lead to biased estimation of regression coefficients and the standard errors of the coefficients and consequently, to misleading test of hypothesis [63]. If the predictors in Eq (15) are considered to have measurement errors, a new approach for the nonlinear mixed-effect models should be developed. However, so far, no algorithms and corresponding computational programs to implement this approach have been available. We are in the process of developing such an appropriate algorithm to deal with this problem, which will certainly be very challenging.

## Appendix 1 Parameters and Matrix Structures for Calculation of Random Effects at Both Plot and Block Levels

At both plot and block levels,

$${\hat{b}}_{i}={\left(u_{i}^{T},u_{i1}^{T},u_{i2}^{T},\ldots ,u_{iM_{i}}^{T}\right)}^{T}\;\left(i=1,\ldots ,M\right)$$

Is (*q*_1_ + *M*_*i*_ *q*_2_) × 1 dimensional augmented random effect vector for the *i* ^*th*^ block.

$$\hat{G}=diag(\psi_{1},\psi_{2},\ldots ,\psi_{2})$$

is (*q*_1_ + *M*_*i*_ *q*_2_) × (*q*_1_ + *M*_*i*_ *q*_2_) dimensional block diagonal positive definite matrix,

$${\hat{C}}_{i}={\left[\begin{array}{ccccc} Z_{i1} & E_{i1} & & & \\ Z_{i2} & & E_{i2} & & \\ \vdots & & & \ddots & \\ Z_{iM_{i}} & & & & E_{iM_{i}} \end{array}\right]}_{n_{i}\times (q_{1}+M_{i}q_{2})}$$

is design matrix. **Z**_*ij*_ and **E**_*ij*_ (*j* = 1,…,*M*_*i*_) are *n*_*ij*_ × *q*_1_ and *n*_*ij*_ × *q*_2_ dimensional matrices of the partial derivatives of the nonlinear function *f*(·) with respect to random effect parameters **u**_*i*_ and **u**_*ij*_, respectively. *M*_*i*_ is the number of plots within the *i* ^*th*^ block. *n*_*ij*_ is the number of observations in the *j* ^*th*^ plot of the *i* ^*th*^ block. *q*_1_ and *q*_2_ are the number of dimensions of **u**_**i**_ and **u**_**ij**_, respectively. **Ψ**_**1**_ and **Ψ**_**2**_ are the variance-covariance matrixes of **u**_**i**_ and **u**_**ij**_, respectively. As mentioned in modeling approach section, the values of ${\hat{u}}_{i}$ in prediction are the same as the estimates obtained in modeling.

## Supporting Information

S1 File Contains the following files: **Table A**, Observed tree attributes of the fitting and validation data sets. **Table B**, Results of performance for the used crown ratio models. **Table C**, Detailed results for the 10 converged mixed-effect models using R/S-Plus *nlme* function.
(RAR)

## References

[1] Soares P, Tomé M (2001) A tree crown ratio prediction equation for eucalypt plantations. Annals of Forest Science 58: 193–202.

[2] Leites LP, Robinson AP, Crookston NL (2009) Accuracy and equivalence testing of crown ratio models and assessment of their impact on diameter growth and basal area increment predictions of two variants of the forest vegetation simulator. Canadian Journal of Forest Research 39: 655–665.

[3] Popoola FS, Adesoye PO (2012) Crown ratio models for tectona ģrandis (Linn. f) stands in Osho forest reserve, Oyo state, Niġeria. Journal of Forest Science 28(2): 63–67.

[4] Assmann E (1970) The Principles of Forest Yield Study. Pergamon Press, Oxford 506 p

[5] Hasenauer H, Monserud RA (1996) A crown ratio model for Austrian forests. Forest Ecology and Management 84: 49–60.

[6] Kershaw JA, Maguire DA, Hann DW (1990) Longevity and duration of radial growth in Douglas-fir branches. Canadian Journal of Forest Research 20: 1690–1695.

[7] Navratil S (1997) Wind Damage in Thinned Stands. In Proceedings of a Commercial Thinning Workshop. October 17–18. Whitecourt, pp 29–36.

[8] Clutter JL, Fotson JC, Pienaar LV, Brister GH, Bailey RL (1983) Timber Management: A Quantitative Approach. John Wiley and sons, New York.

[9] McGaughey RJ (1997) Visualizing Forest Stand Dynamics Using the Stand Visualization System. Proceedings of the 1997 ACSM/ASPRS Annual Convention and Exposition. April 7–10. Seattle, pp 248–257.

[10] Temesgen H, Lemay V, Mitchell SJ (2005) Tree crown ratio models for multi-species and multi-layered stands of southeastern British Columbia. The Forestry Chronicle 81(1): 133–141.

[11] Toney C, Reeves M (2008) Equations to convert compacted crown ratio to uncompacted crown ratio for trees in the interior west. Western Journal of Applied Forestry 24(2): 76–82.

[12] Bragg DC (2001) A local basal area adjustment for crown width prediction. Northern Journal of Applied Forestry 18(1): 22–28.

[13] Sönmez T (2009) Diamater at breast height-crown diameter prediction models for Picea orientalis. African Journal of Agriculture Research 4(3): 215–219.

[14] West PW, Ratkowsky DA, Davis AW (1984) Problems of hypothesis testing of regressions with multiple measurements from individual sampling units. Forest Ecology and Management 7: 207–224.

[15] Calama R, Montero G (2004) Interregional nonlinear height-diameter model with random coefficients for stone pine in Spane. Canadian Journal of Forest Research 34: 150–163.

[16] Pinherio JC, Bates DM (2000) Mixed-Effects Models in S and S-PLUS. Spring-Verlag, New York, NY.

[17] Henderson CR, Kempthorne O, Searle SR, Krosigk CM (1959) The estimation of environmental and genetic trends from records subject to culling. Biometrics 15: 192–217.

[18] Fox J, Ades PK, Bi H (2001) Stochastic structure and individual-tree growth models. Forest Ecology and Management 154: 261–276.

[19] Lindstrom MJ, Bates DM (1990) Nonlinear Mixed Effects Models for Repeated Measures Data. Biometrics 46: 673–687. 2242409

[20] Vonesh EF, Chinchilli VM (1997) Linear and nonlinear models for the analysis of repeated measurements. Marcel Dekker, New York.

[21] Gregoire TG, Schabenberger O, Barrett JP (1995) Linear modeling of irregularly spaced, unbalanced, longitudinal data from permanent plot measurements. Canadian Journal of Forest Research 25: 137–156.

[22] Hall DB, Bailey RL (2001) Modeling and prediction of forest growth variables based on multilevel nonlinear mixed models. Forest science 47(3): 311–321.

[23] Nanos N, Rafael C, Gregorio M, Luis G (2004) Geostatistical Prediction of Height-Diameter Models. Forest Ecology and Management 195: 221–235.

[24] Meng SX, Huang S (2009) Improved Calibration of Nonlinear Mixed-Effects Models Demonstrated on a Height Growth Function. Forest science 55(3): 239–248.

[25] Yang Y, Huang S (2011) Comparison of different methods for fitting nonlinear mixed forest models and for making predictions. Canadian Journal of Forest Research 41(8): 1671–1686.

[26] Zhao D, Kane M, Borders BE (2012) Crown ratio and relative spacing relationships for Loblolly pine Plantations. Open Journal of Forestry 2(3): 107–112.

[27] Rijal B, Weiskittel AR, Kershaw JA (2012) Development of height to crown base models for thirteen tree species of the North American Acadian Region. The Forestry Chronicle 88(1): 60–73.

[28] Raulier F, Lambert M, Pothier D, Ung C (2003) Impact of dominant tree dynamics on site index curves. Forest Ecology and Management 184, 65–78.

[29] Du J, Tang S, Wang H (2000) Update models of forest resource data for subcompartments in natural forest. Scientia silvae sinicae 36(2): 26–32. (In Chinese with English abstract)

[30] Fiala ACS, Garman SL, Gray AN (2006) Comparison of five canopy cover estimation techniques in the western oregon cascades. Forest Ecology and Management 232: 188–197.

[31] O’ Hara KL, Valappil NI (1999) Masam—a flexible stand density management model for meeting diverse structural objectives in multiaged stands. Forest Ecology and Management 118: 57–71.

[32] Sharma M, Parton J (2009) Modeling stand density effects on taper for jack pine and black spruce plantations using dimensional analysis. Forest science 55(3): 268–282.

[33] Reineke PJ (1933) Perfecting a Stand-Density Index for Evenaged Forests. Agricultural Research 46: 627–638.

[34] Yang Y, Huang S, Meng SX, Trincado G, VanderSchaaf CL (2009) A multilevel individual tree basal area increment model for aspen in boreal mixedwood stands. Canadian Journal of Forest Research 39: 2203–2214.

[35] Davidian M, Giltinan DM (1995) Nonlinear Models for Repeated Measurement Data. Chapmanand Hall, New York.

[36] Uzoh FCC, Oliver WW (2008) Individual tree diameter increment model for managed even-aged stands of ponderosa pine throughout the western United States using a multilevel linear mixed effects model. Forest Ecology and Management 256: 438–445.

[37] Spurr SH, Barnes BV (1980) Forest ecology 3rd edn. John Wiley, New York, 527 pp.

[38] Uzoh FCC, Oliver WW (2006) Individual tree height increment model for managed even- aged stands of ponderosa pine throughout the western United States using linear mixed effects models. Forest Ecology and Management 21(1/3): 147–154.

[39] Sánchez-González M, Cañellas I, Montero G (2007) Generalized height- diameter and crown diameter prediction models for cork oak forests in Spain. Investigacián Agraria: Sistemas y Recursos Forestales 16(1): 76–88.

[40] Venables WN, Ripley BD (1999) Modern Applied Statistics with S-PLUS, 3rd ed., Springer-Verlag, New York.

[41] Huuskonen S, Miina J (2007) Stand-level growth models for young Scots pine stands in Finland. Forest Ecology and Management 241(1–3): 49–61.

[42] Pinheiro JC, Bates DM (1995) Approximations to the Loglikelihood Function in the Nonlinear Mixed Effects Model. Journal of Computational and Graphical Statistics 4: 12–35.

[43] Fang Z, Bailey RL (2001) Nonlinear Mixed-Effect Modeling for Slash Pine Dominant Height Growth Following Intensive Silvicultural Treatments. Forest science 47: 287–300.

[44] Calama R, Montero G (2005) Multilevel linear mixed model for tree diameter increment in stone pine (*pinus pinea*): a calibrating approach. Silva Fennica 39(1): 37–54.

[45] Fu L, Sun H, Sharma RP, Lei Y, Zhang H, Tang S (2013) Nonlinear mixed-effects crown width models for individual trees of Chinese fir (*Cunninghamia lanceolata*) in south-central China. Forest Ecology and Management 302: 210–220.

[46] Temesgen H, Monleon VJ, Hann DW (2008) Analysis and comparison of nonlinear tree height prediction strategies for Douglas-fir forests. Canadian Journal of Forest Research 38: 553–565.

[47] Crecente-Campo F, Tomé M, Soares P, Dieguez-Aranda U (2010) A generalized nonlinear mixed-effects height-diameter model for Eucalyptus globulus L. in northwestern Spain. Forest Ecology and Management 259(5): 943–952.

[48] Eerikäinen K (2003) Predicting the height-diameter pattern of planted Pinus kesiya stands in Zambia and Zimbabwe. Forest Ecology and Management 175: 355–366.

[49] Monserud RA, Sterba H (1996) A basal area increment model for individual trees growing in even-and uneven-aged forest stands in Austria. Forest Ecology and Management 80(1–3): 57–80.

[50] Tahvanainen T, Forss E (2008) Individual tree models for the crown biomass distribution of Scots pine Norway spruce and birch in Finland. Forest Ecology and Management 255(3/4): 455–467.

[51] Bella IC (1971) A new competition model for individual trees. Forest science 17: 364–372

[52] Wykoff WR, Crookston NL, Stage AR (1982) User’s guide to the Stand Prognosis model. USDA For. Serv. Gen. Tech. Rep. INT-133.

[53] Sprinz PT, Burkhart HE (1987) Relationships between tree crown, stem and stand characteristics in unthinned loblolly pine plantations. Canadian Journal of Forest Research 17: 534–538.

[54] Ademe P, Río MD, Cañellas I (2008) A mixed nonlinear height-diameter model for Pyrenean oak (*Quercus pyrenaica* Willd.). Forest Ecology and Management 256: 88–98.

[55] Tang S, Li Y, Wang Y (2001) Simultaneous Equations, Errors-in-variable Models, and Model Integration in Systems Ecology. Ecol. Model,142(3), 285–294.

[56] Tang S, Zhang S. 1998 Measurement error models and their applications. J. Biomath. 13, 161–166.

[57] Hoeting JA, Madigan D, Raferty AE, Volinsky CT (1999) Bayesian model averaging—a tutorial. Statistical science 14(4): 382–417.

[58] Omule AY (1980) Personal bias in forest measurement. The Forestry Chronicle 56(5): 222–224.

[59] Gertner GZ (1990) The sensitivity of measurement error in stand volume estimation. Canadian Journal of Forest Research 20: 800–804.

[60] McRoberts RE, Hahn JT, Hefty GJ, Van Cleve JR (1994) Variation in forest inventory field measurements. Canadian Journal of Forest Research 24(9): 1766–1770.

[61] Nicholas NS, Gregoire TG, Zedaker SM (1991) The reliability of tree crown classification. Canadian Journal of Forest Research 21: 698–701.

[62] Ghosh S, Innes JL, Hoffmann C (1995) Observer variation as a source of error in assessments of crown condition through time. Forest science 41(2): 235–254.

[63] Fuller WA (1987) Measurement error models. John Wiley and Sons, New York.

